# ACSL4 as a Potential Target and Biomarker for Anticancer: From Molecular Mechanisms to Clinical Therapeutics

**DOI:** 10.3389/fphar.2022.949863

**Published:** 2022-07-13

**Authors:** Jun Hou, Changqing Jiang, Xudong Wen, Chengming Li, Shiqiang Xiong, Tian Yue, Pan Long, Jianyou Shi, Zhen Zhang

**Affiliations:** ^1^ Department of Cardiology, Chengdu Third People’s Hospital/Affiliated Hospital of Southwest Jiao Tong University, Chengdu, China; ^2^ School of Materials Science and Engineering, Southwest Jiaotong University, Chengdu, China; ^3^ Department of Pharmacy, General Hospital of Western Theater Command, Chengdu, China; ^4^ Department of Gastroenterology and Hepatology, Chengdu First People’s Hospital, Chengdu, China; ^5^ Clinical Medical College, Chengdu University of Traditional Chinese Medicine, Chengdu, China; ^6^ Personalized Drug Therapy Key Laboratory of Sichuan Province, Department of Pharmacy, Sichuan Academy of Medical Science and Sichuan Provincial People’s Hospital, School of Medicine, University of Electronic Science and Technology of China, Chengdu, China

**Keywords:** acyl-CoA synthetase long-chain family, anticancer biomarker, glucolipid metabolism, ferroptosis, arachidonic acid

## Abstract

Cancer is a major public health problem around the world and the key leading cause of death in the world. It is well-known that glucolipid metabolism, immunoreaction, and growth/death pattern of cancer cells are markedly different from normal cells. Recently, acyl-CoA synthetase long-chain family 4 (ACSL4) is found be participated in the activation of long chain fatty acids metabolism, immune signaling transduction, and ferroptosis, which can be a promising potential target and biomarker for anticancer. Specifically, ACSL4 inhibits the progress of lung cancer, estrogen receptor (ER) positive breast cancer, cervical cancer and the up-regulation of ACSL4 can improve the sensitivity of cancer cells to ferroptosis by enhancing the accumulation of lipid peroxidation products and lethal reactive oxygen species (ROS). However, it is undeniable that the high expression of ACSL4 in ER negative breast cancer, hepatocellular carcinoma, colorectal cancer, and prostate cancer can also be related with tumor cell proliferation, migration, and invasion. In the present review, we provide an update on understanding the controversial roles of ACSL4 in different cancer cells.

## Introduction

Cancer is a serious chronic disease which becomes the key causes of death and disability in the world. An analysis of the global burden of cancer 1990–2019 showed that more than 10 million people died of cancer in 2019, roughly double the number in 1990 (Lin et al., 2021). Using data updated in 2020 from the International Agency for Research on Cancer, it is estimated that there are 19.3 million new cancer cases and nearly 10 million cancer patients’ death worldwide in 2020 (Sung et al., 2021).

It is well-known that early-prevention, early-diagnosis, and early-treatment is the most effective strategy for cancer control. Biomarkers refer to substances characterized by production or abnormal production of malignant tumor cells, or substances produced by the host in response to tumor stimulation, which can reflect the occurrence and development of tumor and monitor tumor response to treatment. Commonly, tumor markers exist in the tissues, body fluids, and excreta of tumor patients and can be detected by immunological, biological, and chemical methods. Consequently, exploring additional molecular markers to surveil tumors emergence and metastasis has become a hot topic for scientists.

Fatty acids are one of the main energy sources in mammals and play essential roles in cell growth and metabolism. They are involved in cell membrane structure, energy metabolism, and cellular signaling pathways to maintain cellular physiological functions. The dysregulated fatty acids metabolism causes excessive synthesis and catabolism, leading to various diseases, such as type 2 diabetes, cardiovascular diseases, liver diseases, neurodegenerative diseases, and cancers (Hosseini et al., 2020). Acyl-CoA synthetase long-chain family 4 (ACSL4) is a key enzyme that catalyzes long-chain fatty acids activation, and abnormal expression of ACSL4 is closely related to various biological responses, including steroidogenesis, inflammation response, cell death, immune activation response, and so on (Liao et al., 2022). Specifically, ACSL4 participates in ferroptosis, a promising target for tumor therapeutics. Moreover, previous studies found that ACSL4 had certain effects on cancer progression, recurrence, and prognosis, and was expected to become an available tumor biomarker and therapeutic target.

This review demonstrates the latest progress in the roles of the ACSL4 in different tumors. First, we display the structure and function of the ACSL4. Second, we explore the effect of the ACSL4 in the pathological mechanisms involved with tumors. Third, we discuss the relationship between the ACSL4 and different tumors. Understanding the exact role of ACSL4 in cancer and the molecular mechanism involved would provide ideas for finding new targets for cancer diagnosis and treatment and developing new strategies for therapy.

## Acyl-CoA Synthetase Long-Chain Family 4

Long chain fatty acids (carbon chain length >12) are important nutrients, which can be used as cellular fuel sources, membrane lipid components, protein post-translational modification (PTM), signal transduction pathways, energy storage within adipose tissue, and precursors of bioactive lipid mediators (Nakamura et al., 2014). Firstly, long chain fatty acids combine with fatty acid transport proteins (FATP) to transport into target cells. Then, free long chain fatty acids converted to their respective acyl-CoA forms and catalyzed by ACSL. Among the ACSL family enzymes in mammals, ACSL4 prefers to catalyze several polyunsaturated fatty acids (PUFAs), such as arachidonic acid (AA) and eicosapentaenoic acid (EPA). The PUFAs are precursors of bioactive lipid mediators, and the unique feature of ACSL4 suggests to be participated in various pathophysiological events, including lipid metabolism, ferroptosis, and immune response. Recent studies have shown that ACSL4 expression changes in a variety of cancers, and targeting at ACSL4 could affect tumor progression, suggesting that ACSL4 may be a potential tumor marker and therapeutic target.

### Lipid Metabolism

Acyl-CoA synthetase long-chain family (ACSL) is a key enzyme responsible for lipid metabolism *in vivo*, mainly catalyzing the formation of 12–20 carbon chain length fatty acids (Kuwata and Hara, 2019). ACSL in mammals consists of five ACSL isoenzymes (ACSL1, ACSL3, ACSL4, ACSL5, and ACSL6), which have specific tissue localization and different functions (Wu et al., 2009; Lopes-Marques et al., 2013; Gao et al., 2016; Zhao et al., 2019; Nan et al., 2021). ACSL1 is highly expressed in major energy metabolism tissues such as fat, liver and muscle, functioning with fatty acid intake (Suzuki et al., 1990). ACSL3 is primarily located in the brain, prostate, and muscle and is responsible for activating monounsaturated fatty acids (MUFA), thereby competitively inhibiting PUFA-induced ferroptosis (Fujino et al., 1996; Teodoro et al., 2017; Ubellacker et al., 2020). ACSL5 is elevated in brown adipose tissue, small intestine and liver (Mashek et al., 2006). Moreover, ACSL6 is located in the brain and muscle tissues, which is responsible for the activation of docosahexaenoic acid (DHA) (Fernandez et al., 2018).

In 1997, a novel acyl-CoA synthase based on arachidonic and eicosatetraenoic acids was reported in PNAS (Kang et al., 1997). It was named ACSL4 and was found in adrenal gland, epididymis, brain, seminal vesicles, lungs, ovaries, liver, and many other tissues, with the adrenal gland being the most abundant. Like other mammalian ACS, ACSL4 consists of five regions: an NH2 terminus, luciferase-like regions 1 and 2, a linker connecting the two luciferase-like regions, and a COOH terminus. The amino acids at the luciferin-like region 2 and COOH terminal are highly identical in the ACS family, suggesting that these two regions are critical for the catalytic reaction of ACSL. ACSL4 lacks 50 amino acids corresponding to the NH2 which may cause the different response in fatty acids preference among ACSLs (Kang et al., 1997). The subcellular localization of ACSL4 is mainly in endosomes (Liu et al., 2014) and peroxisomes (Lewin et al., 2001) in the secretory pathway (Ansari et al., 2017). Moreover, ACSL4 transfers to the plasma membrane (Küch et al., 2014) and the endoplasmic reticulum regions in contact with the mitochondria, named mitochondrial associated membranes, which is responsible for fatty acids synthesis and β-oxidation (Tang et al., 2018).

The substrate specificities of the ACSL enzymes significantly differ among the five isozymes, and in particular, ACSL4 prefers PUFAs, such as AA and EPA as its substrate (Kang et al., 1997; Kuwata and Hara, 2019). ACSL4 mainly catalyzed the long chain PUFAs (including arachidonic acid 20:4 and adrenic acid 22:4) to CoA-PUFAs. These products are then esterized into phospholipids by multiple lysophosphatidylcholine acyltransferase (LPCAT), facilitating the incorporation of intracellular long-chain PUFAs into lipids membrane structures (Jiang et al., 2021). In cancer cells, the uptake and metabolism of fatty acids are often dysregulated. Fatty acid activation is a key step that allows these biomolecules to enter cellular metabolic pathways such as mitochondrial β-oxidation to produce ATP or adipogenesis pathways. Enhanced expression of particular ACSL4 was confirmed to be a feature of some more aggressive cancers and may contribute to the oncogenic phenotype (Radif et al., 2018).

### Ferroptosis

Ferroptosis is a new type of cell death characterized by a large iron-dependent accumulation of lethal lipids and reactive oxygen species (ROS), which is different from apoptosis, necrosis, and autophagy, first proposed by Dixon in 2012 (Dixon et al., 2012). Commonly, Ferroptosis is characterized by three basic features 1) Oxidation of PUFA (containing membrane phospholipids); 2) Iron utilizing related REDOX activity; 3) Loss of repair ability of lipid hydroperoxide (LOOH). Specifically, the cell death process is accompanied by the accumulation of a large number of irons, and lipid peroxidation, and changes in some genes that regulate iron homeostasis and lipid peroxidation metabolism. In the microscopic structure of cells, there are smaller mitochondria than normal cells, and the mitochondrial membrane shrinks, while the mitochondrial crest decreases or disappears, and the outer membrane is broken, but the morphological changes in the nucleus are not obvious (Lei et al., 2022). Up to now, ferroptosis has been linked to a variety of human diseases, such as ischemic organ injury, neurodegeneration and cancer (Su et al., 2020; Li and Huang, 2022).

**FIGURE 1:**
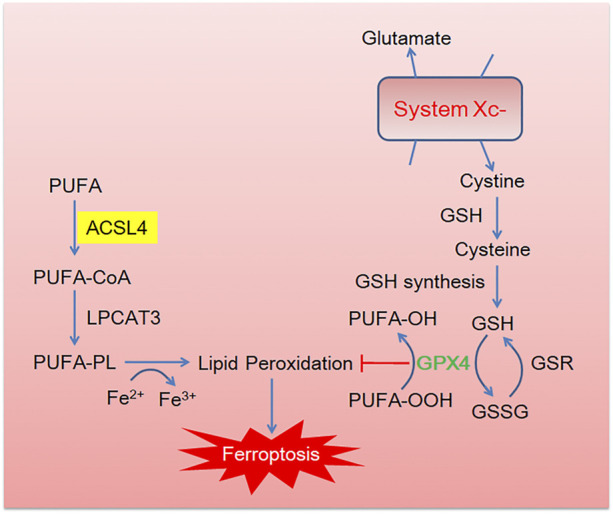
The brief description of canonical ferroptosis pathway. Cystine enters into cells through the cystine/glutamic acid reverse transporter (System Xc-) and then reduces to cysteine in the glutathione (GSH). GSH acts as a cofactor of glutathione peroxidase 4 (GPX4) to promote the reduction of phospholipid hydroperoxides (PLOOHs) to corresponding alcohols (PLOHs) in cells. Essential lipid peroxidase acyl-CoA synthase long chain family member 4 (ACSL4) and lysophosphatidylcholine acyltransferase (LPCAT) activate PUFA into PUFA-CoA and PUFA-PL, respectively, leading to lipid peroxidation.

As shown in Figure 1, canonical pathway of ferroptosis includes lipometabolic disturbance, glutathione (GSH)-glutathione peroxidase 4 (GPX4) exhaustion, cystine deprivation and abnormal iron metabolism. As for anti-ferroptosis pathway, ferroptosis suppressor protein 1(FSP1)- coenzyme Q (CoQ10)- Nicotinamide Adenine Dinucleotide Phosphate (NADPH) and methovalerate pathway are well-studied (Stockwell, 2019). Importantly, the phospholipid acyl chain remodeling pathway is the key process to ferroptosis. Specifically, AA and other PUFAs from lipid bilayer can be metabolized into AA-CoA and AA-PL by ACSL4 and LPCAT, respectively.

Ferroptosis is tightly associated with lipid peroxidation, in which enzymes that regulate PUFA metabolism, especially ACSL4. ACSL4 activates PUFAs and sensitizes cancer cells to ferroptosis in immunotherapy-related settings. Exogenous oxygen radicals generated by photodynamic therapy could peroxidize PUFAs (accompanied with higher expression of ACSL4) and promote ferroptosis to cancer treatment (Shui et al., 2021). Suppression ACSL4 by genetic or pharmacological inhibition could act as a specific anti-ferroptotic rescue pathway (Kagan et al., 2017). Studies showed that ionizing radiation induced ferroptosis in cancer cells by inducing ROS and activating ACSL4 (Lei et al., 2020). Therefore, ACSL4 could become a promising drug target for certain tumor treatment *via* ferroptosis pathway.

### Immune Response

Commonly, ACSL4 has a wide range of biological effects, and has been reported to be involved in inflammation, steroid production, cell death, and so on. ACSL4 is found in adrenal zonulate and reticulum zonules, luteal and interstitial luteal cells of ovary, and interstitial cells of testis, participating in various immune responses.

**FIGURE 2:**
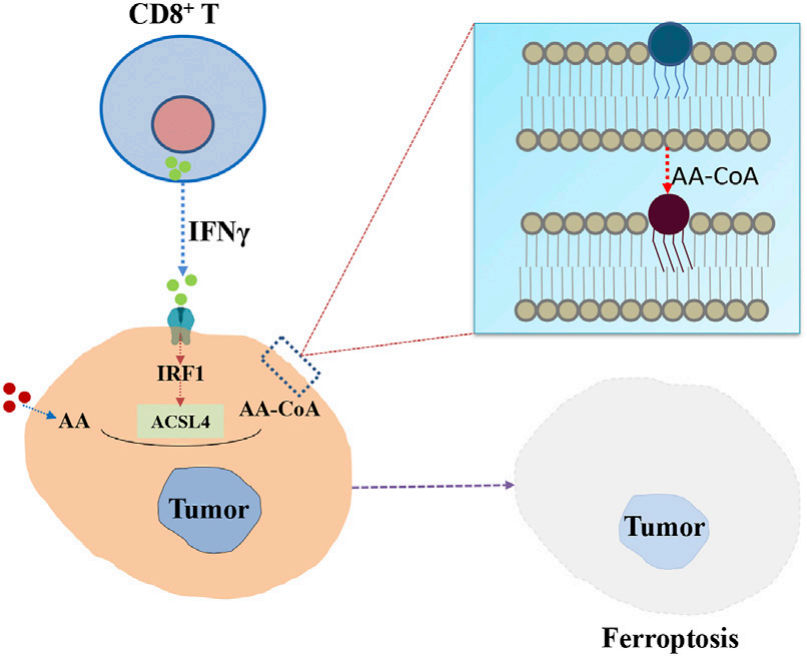
The potential role of ACSL4 in specific immune responses mediated by CD8^+^ T cells. IFN-γ secreted by CD8^+^ T cells stimulates ACSL4 and alters tumor cell lipid patterns, thereby increasing the binding of AA in C16 and C18 acyl-chain phospholipids. Lipid peroxidation then leads to ferroptosis of tumor cells.

It is well-known that ACSL4 can activate AA to initial the production processes of prostaglandin and leukotrienes synthesis. Then nonspecific immune response is activated through the release of inflammatory medium. Recently, Liao et al. (2022) demonstrated ACSL4 could play an essential role in CD8^+^ T cell (CTL) mediated specific immune response, correlating with increased immunosurveillance and responding to checkpoint blockade (Kepp and Kroemer, 2022). Specifically, Figure 2 shows that interferon-γ (IFN-γ) secreted by CD8^+^ T cells, together with AA, can promote ACSL4-mediated ferroptosis, which is a mode of action for CTL-mediated tumor killing. IFN-γ stimulates ACSL4 and changes the lipid pattern of tumor cells, thereby increasing the binding of AA to C16 and C18 acyl-chain phospholipids. Common C16 and C18 fatty acids palmitic and oleic acid in blood promote IFN-γ + AA induced ferroptosis of ACSL4-associated tumors. Interestingly, low doses of AA enhanced tumor ferroptosis and enhanced spontaneous and immune checkpoint blockade (ICB) -induced antitumor immunity. ACSL4 activates PUFAs and sensitizes cancer cells to ferroptosis in immunotherapy-relevant settings. Late study found genetic deletion of ACSL4 could result in an impaired antitumor CD8^+^ T cell responses (Drijvers et al., 2021). These findings provide insights into how the metabolic and immune milieu could be used to promote ferroptosis (Friedmann Angeli et al., 2022).

## Acyl-CoA Synthetase Long-Chain Family 4 and Cancer

Recently, reprogramming of cellular energetics is a hallmark of cancer attracts researchers’ attention (Pavlova and Thompson, 2016). Fatty acids, on the one hand, provide energy for cells by replacing glucose with β-oxidation (Park et al., 2016; Manley et al., 2017) and, on the other hand, drive phospholipid anabolic metabolism, which increases membrane biosynthesis and induces the production of signaling proteins in cancer cells (Röhrig and Schulze, 2016; Tan et al., 2018).

Among the five mammalian ACSL family members, ACSL1 and ACSL3 are involved in facilitating cancer progression, while ACSL5 participates in the pro-apoptotic sensing of cells, acting as a tumor suppressor (Quan et al., 2021). However, as shown in Table 1 ACSL4 could play controversial roles being as a tumor accelerator or tumor suppressor depending on the specific cancer types and tissue environment. ACSL4 activate long chain fatty acids to initiate a number of intracellular lipid metabolic pathways (Kuwata and Hara, 2019; Rossi Sebastiano and Konstantinidou, 2019). Emerging evidences showed that dysregulated expression of ACSL4 was tightly associated with various diseases and especially with cancers (Dattilo et al., 2019; Orlando et al., 2019; Rossi Sebastiano and Konstantinidou, 2019). Mechanisms of ACSL4 involvement in tumor development may include iron-dependent, non-apoptotic, and cell death pathways (Doll et al., 2017), drug resistance caused by metabolic recombination (Orlando et al., 2019), arachidonic acid-dependent tumorigenesis (Orlando et al., 2012), steroid production (Wang et al., 2019) and activation of intracellular pro-cancer signaling pathways (Wu et al., 2015). In fact, the predictive value of ACSL4 in several cancers has been revealed by a multiple databases analysis (Yu et al., 2022). Specifically, ACSL4 is positively correlated with immune infiltration in the tumor microenvironment, which is intensively related to prognosis in breast invasive carcinoma and skin cutaneous melanoma. Additionally, ACSL4 point mutations and ACSL4-associated hypomethylation usually indicated poor prognosis in generalized carcinoma (Yu et al., 2022). Below, we would like to introduce the relationship between ACSL4 and different cancers, respectively.

**TABLE 1:** Impact of ACSL4 expression in different cancer types.

Target	Cancer type		Effect	References
ACSL4	Breast cancer	ER (+)	Overexpression is a predictor of good prognosis of breast cancer	Ragab et al. (2018), Dinarvand et al. (2020)
		ER (−)	Overexpression increases the aggressiveness of breast cancer	Maloberti et al. (2010), Monaco et al. (2010), Yu et al. (2022)
	Lung cancer		Low expression has a poor prognosis in lung adenocarcinoma	Zhang et al. (2021)
	Colorectal cancer		KRAS mutant colorectal cancer cell line shows significant upregulation of ACSL4	Park et al. (2018)
	Hepatocellular carcinoma		OS and DFS time of HCC patients with high ACSL4 expression are significantly shortened	Calvisi et al. (2011), Matter et al. (2014), Wang et al. (2020)
	Cervical cancer		High expression of ACSL4 promotes the sensitivity of cervical cancer cells to chemotherapy	Xiaofei et al. (2021)
	Prostate cancer		Downregulation of ACSL4 inhibits the proliferation, migration, invasion and growth of non-AR dependent prostate cancer cells	Baron et al. (2004), Currie et al. (2013)

ER, estrogen receptor; OS, overall survival; DFS, disease-free survival.

### Acyl-CoA Synthetase Long-Chain Family 4 and Breast Cancer

Breast cancer is one of the most dangerous diseases threatening women’s/men’s health. New estimates found that nearly 440,000 patients died of breast cancer each year (Wang et al., 2017). Unfortunately, breast cancer still has no effectively predictive and prognostic biomarkers. Recent evidences showed that inducing ferroptosis may enhance the efficacy of cancer therapy. ACSL4 have been well established as the positive regulator of ferroptosis and could be served as a novel predictive/prognostic breast cancer biomarker.

Recently, clinical studies demonstrated that higher ACSL4 expression was related with enhanced sensitivity to neoadjuvant chemotherapy in breast cancer, leading to a better overall survival (Sha et al., 2021). Moreover, nuclear protein Ki-67, whose function is closely related to mitosis, is considered as a marker of cell proliferation levels (Denkert et al., 2015). Several studies have shown that high expression of Ki-67 was accompanied with higher risk of recurrence, poor prognosis, and lower survival time. Interestingly, ACSL4 was found be negatively correlated with Ki-67 expression in breast cancer patients (Ragab et al., 2018). As for cell studies showed targeting ACSL4 could improve the response to irradiation and inhibit migration activities (Kwon et al., 2021). Negar Dinarvand found the prognostic significance of the expression of ACSL4 in breast cancer patients, and it was closely correlated with tumor suppressor p53 (Dinarvand et al., 2020). In conclusion, these results suggested that higher expression of ACSL4 was a predictor of better prognosis of breast cancer.

Nevertheless, there were also evidences that ACSL4 overexpression increased the aggressiveness of breast cancer. ACSL4 expression was significantly higher in breast cancer tissues than that in adjacent tissues. ACSL4 expression was 0.386 times higher on average in p53-positive patients than in p53-negative individuals. Additionally, in both breast tumor cells and animal models, the use of PRGL493, a chemical inhibitor of ACSL4 impeding *de novo* steroid synthesis, could block cell proliferation and tumor growth, and promote the sensitivity of tumor cells to chemotherapeutic and hormonal treatment (Castillo et al., 2021). It is well-known that estrogen receptor (ER) negative breast cancer is less sensitive to chemotherapy, more likely to relapse, leading to poor prognosis. RT-PCR detections confirmed that only 2 of the 19 (10.5%) ER positive breast cancer cell lines existed ACSL4 mRNA expression, and ACSL4 mRNA was broadly expressed in 20 of 31 (64.5%) ER negative cell lines (Monaco et al., 2010). This indicated that ACSL4 mRNA expression may be correlated with more aggressive ER negative breast cancer. Moreover, Maloberti et al. (2010) found that ACSL4 was significantly upregulated in highly aggressive MDA-MB-231 breast cancer cells and played a key role in enhancing its aggressiveness. Moreover, ACSL4 was found to be more expressed in estrogen receptor (ER)-negative cancers, such as quadruple negative breast cancer (QNBC), than that in ER-positive cancers (Yen et al., 2017). ACSL4 levels were negatively correlated with hormone/growth factor receptor expression and positively correlated with the most aggressive form of QNBC (Huang et al., 2020). According to these studies, lower ACSL4 expression points more beneficial prognosis, and ACSL4 may serve as a promising prognostic biomarker for invasive breast cancer. The prognosis of breast cancer predicted by ACSL4 is related to the type of breast cancer. In ER receptor negative patients, higher ACSL4 expression could predict the poor prognosis.

ACSL4 serves not only as a prognostic biomarker but also as a therapeutic target. ACSL4, as a crucial molecule that regulates ferroptosis, is preferentially expressed in a group of basal-like breast cancer cell lines, and its expression appears to be closely associated with sensitivity to RSL3-induced ferroptosis (Doll et al., 2017). In addition, ACSL4 may be an effective therapeutic target for the regulation of multiple transporters associated with anti-cancer resistance through the mammalian target of rapamycin (mTOR) pathway, thereby restoring drug sensitivity in breast cancer with poor prognosis (Orlando et al., 2019). Thus, ACSL4 may serve as a valuable biomarker for breast cancer as well as a target for therapy in the way of promoting ferroptosis and drug sensitivity.

### Acyl-CoA Synthetase Long-Chain Family 4 and Lung Cancer

Lung cancer has a high mortality rate, which is one of the common type of cancer (Siegel et al., 2017). The mechanism of lung cancer progression and the exploration of treatment strategies are still important research topics (Liu et al., 2018). Adenocarcinoma of lung is the dominant pathological types, accounting for approximately 30% of new diagnosed lung cancer worldwide (Barta et al., 2019). Due to early metastasis and recurrence, the 5-year survival rate of lung adenocarcinoma is less than 30% (Lin et al., 2019).

The Cancer Genome Atlas (TCGA) analyzing and clinical samples verification showed that ACSL4 was frequently downregulated in lung adenocarcinoma (Zhang et al., 2021). Furthermore, Kaplan-Meier survival analysis showed that patients with lower ACSL4 expression had worse progression-free survival and overall survival than patients with higher ACSL4 expression (Zhang et al., 2021). Gene set enrichment analysis found the increasing expression of ACSL4 was related with ferroptosis-related proteins. *In vivo* experiments demonstrated that knockdown of ACSL4 could improve the ability of tumor invasiveness and inhibit ferroptosis, while ACSL4 overexpression represented the opposite effects (Zhang et al., 2021). Additionally, previous studies found free fatty acid metabolism could affect ACSLs expression and cell sensitivity to ferroptosis (Magtanong et al., 2019). High-fat intervention on cancer cells could inhibit erastin-induced ferroptosis by decreasing the expression of ACSL4. It was reported that the nuclear paraspeckle assembly transcript 1 (NEAT1, a long non-coding RNA) was regarded as a novel target for diagnosis and therapy in human tumors, and higher NEAT1 expression was together with worse survival in cancer patients (Dong et al., 2018). Recent study found that the NEAT1 could target binding with ACSL4 and downregulate the expression of ACSL4, resulting in decreased sensitivity of non-small cell lung cancer (NSCLC) cells to ferroptosis (Wu and Liu, 2021). Although most studies revealed that the low expression of ACSL4 in lung cancer was a biomarker of adverse outcomes, some studies showed that the upregulation of *ACSL4* gene may be one of the causes of lung cancer. The expression of *ACSL4* gene was significantly upregulated in the tobacco exposure group compared with the non-smoking group (Xing et al., 2015).

### Acyl-CoA Synthetase Long-Chain Family 4 and Colorectal Cancer

Colorectal cancer is seen with increasing frequency, being one of the most common causes of cancer mortality worldwide. The important reasons of death should be resulted from late diagnosis and recurrence or metastasis of tumor cells and new therapeutic strategies are urgently needed (Rajalingam et al., 2007; Prior et al., 2012). As we all-known that KRAS mutations were one of the most prominent oncogenes in colorectal cancer. Studies have found that mutations in KRAS were found in 30%–50% of colorectal cancers (Liu et al., 2011). In addition, patients with KRAS mutations represented a poor prognosis compared to all other patients (Inoue et al., 2012).

Here, we focus on the potential roles of ACSL4 in colorectal cancer. Dysregulated lipid metabolism resulted in cancer progression and previous studies indicated that ACSLs were essential for lipid regulation. Systematic analysis and *in vitro* experiment confirmed that high expression of ACSL4 predicted a worse prognosis in colorectal cancer and downregulating ACSL4 could reduce cell proliferation and invasion (Chen et al., 2016). Metabolic reprogramming is a prominent feature of cancer. ACSL/stearoyl-CoA desaturase (ACSL1/ACSL4/SCD) metabolic network disorders, resulting in elevation of acylcarnitines, downregulation of polyunsaturated fatty acids (PUFA), and upregulation of monounsaturated fatty acids (MUFA), could cause invasion and poor prognosis in colorectal cancer (Sánchez-Martínez et al., 2017). Unlike the partly positive roles of ACSL4 in breast cancer, higher ACSL4 expression resulted in colorectal cancer cells proliferation and migration accompanied by a shorter survival time in colorectal cancer patients (Sánchez-Martínez et al., 2017). Moreover, Park et al. (2018) treated colorectal cancer cells with bromelain and analyzed the expression level of genes involved in cell signaling pathway. The results showed that compared with KRAS wild-type colorectal cancer cell line, KRAS mutant colorectal cancer cell line showed significant upregulation of ACSL4. Specific shRNA knockout of ACSL4 could inhibit erastin-induced ferroptosis in KRAS mutant DLD-1 cells, indicating that ACSL4 was a key regulatory molecule for bromelain to effectively inhibit KRAS mutant colorectal cancer by stimulating ferroptosis (Park et al., 2018). The result showed that bromelain could significantly inhibit the growth and proliferation of colorectal cancer cells, but the downstream molecular mechanism was unknown.

### Acyl-CoA Synthetase Long-Chain Family 4 and Hepatocellular Carcinoma

Hepatocellular carcinoma (HCC) has become the second leading cause of cancer mortality worldwide, resulting in 819,000 deaths per year (Fitzmaurice et al., 2019). Furthermore, HCC is the main pathological pattern among all primary liver malignancies with 90% composition (Jemal et al., 2011). As we all-known, lipid metabolic reprogramming is tightly related with HCC proliferation, migration and invasion. Interestingly, ACSL4, a member of acyl-CoA synthetases family, is frequently upregulated in HCC and associated with poor prognosis. Furthermore, ACSL4 isoforms could be used to divided HCC, cholangiocarcinoma (CCA) and hepatic metastases (Ndiaye et al., 2020). Functionally, ACSL4 knockdown could induce decreased cell proliferation, whereas upregulation ACSL4 expression could activate tumor formation *in vitro* and *in vivo*. The molecular mechanisms could be related with microRNAs, PTMs, oncogene activation, and so on. Specifically, evidences showed that microRNAs miR-211-5p was involved in the HCC progression and prognosis, while ACSL4 was a direct downstream target of miR-211-5p. And miR-211-5p could inhibit the malignant phenotype by reducing the expression of ACSL4 protein (Qin et al., 2020). Compared with normal tissues, the relative expressions of miR-211-5p in HCC tissues and cell lines were significantly downregulated, and the upregulation of miR-211-5p *in vitro* continuously inhibited. Moreover, ACSL4 could upregulate the master lipogenesis regulator sterol regulatory element binding protein 1 (SREBP1) (Chen et al., 2021) and stabilize the oncoprotein c-Myc, resulting in HCC prognosis (Chen et al., 2020). Glycosylation was a common PTM in cancer and immunoprecipitation test demonstrated that ACSL4 protein could be O-GlcNAcylated to maintain protein stability and continuously promote cancer cell proliferation (Wang et al., 2020). Additionally, autophagy dysfunction is a crucial event in the progression of HCC, accounting for increasing cell proliferation and invasion (Calvisi et al., 2011; Ho et al., 2012; Matter et al., 2014). Specifically, the level of mTOR phosphorylation significantly increased when ACSL4 was overexpressed, and decreased when ACSL4 was downregulated. Moreover, rapamycin treatment saved the role of overexpression of ACSL4 in promoting cell growth and inhibiting cell apoptosis.

Moreover, clinical studies have shown that ACSL4 expression level was directly related to HCC prognosis (Sun and Xu, 2017). Oncomine database and TCGA databases were used to explore the relationship between the expression of ACSL4 mRNA in HCC and its prognosis. The results showed that the expression of ACSL4 mRNA in HCC tissues was significantly higher than that in normal tissues. Survival analysis showed that the overall survival and disease-free survival time of HCC patients with high ACSL4 expression were significantly shortened (Cheng et al., 2009; Iavarone et al., 2011). However, ACSL4 could play a positive role in sorafenib-resistant patients with HCC. Sorafenib is the first-line HCC treatment agent and there exists no effective biomarkers to predict sorafenib response sensitivity (Marisi et al., 2018). Recently, studies showed ACSL4 was positively correlated with the efficacy of sorafenib through cell experiment and clinical study. Cell experiments found the expression of ACSL4 protein was negatively related with half maximal inhibitory concentration (IC50) values of sorafenib in hepatoma cell line. Clinical study confirmed the expression of ACSL4 in excised HCC tissue was positively related with sorafenib reaction (Feng et al., 2021). Taken together, ACSL4 could be an essential prediction factor for sorafenib sensitivity in HCC.

### Acyl-CoA Synthetase Long-Chain Family 4 and Cervical Cancer

Cervical cancer is one of the key life-threating disease among women worldwide (Schiffman et al., 2007; Crosbie et al., 2013; Manini and Montomoli, 2018). Recently, researcher found oleanolic acid (OA), a substance obtained from the leaves, fruits, and rhizomes of plants, could significantly reduce the volume of cervical cancer in mice, but the mechanism is unclear (Xiaofei et al., 2021). Interestingly, ACSL4 was highly expressed in cervical cancer cells treated with OA. Using siRNA to inhibit the expression of ACSL4 in cervical cancer cells, the suppression effect of OA on cell proliferation and viability was cancelled (Xiaofei et al., 2021). These results suggest that OA could promote ACSL4-dependent ferroptosis and may be a potential therapeutic approach for cervical cancer. Moreover, previous studies have clarified that circular RNA (circRNA) could participate in inhibiting tumorigenesis and tumor progression. Ou et al. (2022) found circular RNA (CircLMO1) could inhibit cervical cancer proliferation by activating ACSL4-induced ferroptosis, and could be a promising anti-cancer biomarker for cervical cancer. As for chemotherapy treatment of cervical cancer, ACSL4-mediated ferroptosis could play an important role in propofol synergistic anticancer effects with paclitaxel (Zhao et al., 2022).

### Acyl-CoA Synthetase Long-Chain Family 4 and Prostate Cancer

Prostate cancer is one of the most frequent cancer threat to men’s health (Jemal et al., 2008). Surgery or drugs that target androgen receptor (AR) signaling served as the first-line therapy (Harris et al., 2009). As is well-known that castration resistance is an essential reason for poor efficacy of prostate cancer. Studies have found that fatty acid metabolism dysregulated was highly associated with the maintenance of high proliferation rate and tumor growth of prostate cancer cells (Baron et al., 2004; Currie et al., 2013).

Recently, ACSL4, a rate-limiting enzyme, functioning as the conversion of long chain fatty acids into activated fatty acid has sparked great interests of researchers (Monaco et al., 2010; Wu et al., 2015). Studies have shown that AR, as a transcription inhibitor, could bind to the ACSL4 promoter region and inhibit its transcription. Inhibition of androgen-AR signaling, significantly increased ACSL4 levels. Downregulation of ACSL4 significantly inhibits the non-AR dependent prostate cancer cells proliferation, migration and invasion (Ma et al., 2021). Moreover, docetaxel resistance is a key problem in clinical therapy of metastatic prostate cancer and the mechanism is unclear. Researchers found lncRNAs NEAT1 could promote docetaxel-resistant prostate cancer cells proliferation and invasion by sponging miR-204-5p and miR-34a-5p, leading to an increasing expression of ACSL4 (Jiang et al., 2020). And inhibitor targeting to ACSL4 could reduce prostate cancer growth, therapeutic resistance and steroidogenesis (Castillo et al., 2021).

## Summarize and Prospective

In this article, we introduce the cell localization, structure and function of ACSL4, and mainly summarize the evidences of ACSL4 as a potential biomarker and therapeutic target in many cancer types. ACSL4 could activate fatty acids by adding CoA and abnormal expression of ACSL4 was reported in several cancers and may affect prostaglandin biosynthesis, fatty acid β-oxidation, ferroptosis, and phosphatidyl chain remodeling. ACSL4 behaves as a crucial regulator in lipid metabolism, ferroptosis, and immune response, which contributes to its tight association with the onset and progression of various cancers. Due to different cancer types or different subtypes of the same cancer, ACSL4 showed different effects in promoting tumor proliferation or inhibiting cancer cell growth. Specifically, the expression of ACSL4 is significantly upregulated in multiple types of cancer, including breast ER negative, colorectal, and prostate, while it becomes downregulated in other cancers (breast cancer ER positive, lung cancer and cervical cancer). Commonly, in ACSL4 high expressing cancers (e.g., colon or ovarian cancer), increased expression of ACSL4 typically predicts unfavorable outcome/prognosis, while in the ACSL4 low expressing cancers (e.g., liver cancer), decreased expression of ACSL4 is corelated to unfavorable outcome/prognosis. In this context, ACSL4 may be capable of identifying abnormalities to serve as risk, diagnostic or prognostic markers as well as therapeutic targets in a wide range of cancers.

However, the Janus-faced role of ACSL4 in cancers due to the diverse pathophysiology of cancers and the heterogeneous nature of tumors, make the lack of consistency becomes a problem to evaluate the indicative effect of biomarker ACSL4. This may be associated with the different roles of ACSL4 in distinct cancers. Specifically, activation of ACSL4 may play a role in lipid metabolism reprogramming, providing an efficient supply for tumor survival, or inducing antitumor effects leading to tumor death. However, we cannot accurately evaluate whether ACSL4 has more benefits than harms in specific antitumor responses. Although ACSL4 is the activator of ferroptosis in distinct cancers, whether ferroptosis/ACSL4 benefit to improve the outcome of tumors need careful identification. On the one hand, the response to ferroptosis is regulated by a complex network of epigenetic, post-transcriptional modifications, and post-translational modifications. Targeting those pathways that regulate ferroptosis in tumor cells is an emerging antitumor strategy because malignant tumor cells often rely on oncogenic and/or survival signals, making them particularly vulnerable to ferroptosis. On the other hand, ferroptotic injury can trigger inflammation-related immunosuppression in the tumor microenvironment, thereby facilitating tumor growth. Therefore, ACSL4 could play complex roles in tumor promotion and tumor suppression when analyzing different tumors.

As we all-known each candidate biomarker has their own the limitations. Considering the tumor heterogeneity and unique micro-environment, strategy with combinatorial approaches for different tumor markers could be a more accurate way to predict tumor prognosis or therapeutic effect. Therefore, a single biomarker is not reliable for decision making, while a combination of biomarkers and/or algorithms supported by multiple methods will be more successful.
